# Large scale genotype‐ and phenotype‐driven machine learning in Von Hippel‐Lindau disease

**DOI:** 10.1002/humu.24392

**Published:** 2022-05-10

**Authors:** Andreea Chiorean, Kirsten M. Farncombe, Sean Delong, Veronica Andric, Safa Ansar, Clarissa Chan, Kaitlin Clark, Arpad M. Danos, Yizhuo Gao, Rachel H. Giles, Anna Goldenberg, Payal Jani, Kilannin Krysiak, Lynzey Kujan, Samantha Macpherson, Eamonn R. Maher, Liam G. McCoy, Yasser Salama, Jason Saliba, Lana Sheta, Malachi Griffith, Obi L. Griffith, Lauren Erdman, Arun Ramani, Raymond H. Kim

**Affiliations:** ^1^ Department of Medicine, Division of Medical Oncology University Health Network Toronto Ontario Canada; ^2^ Toronto General Hospital Research Institute University Health Network Toronto Ontario Canada; ^3^ Department of Medicine, Division of Oncology, Washington University School of Medicine Washington University St. Louis Missouri USA; ^4^ McDonnell Genome Institute Washington University School of Medicine Missouri St. Louis USA; ^5^ International Kidney Cancer Coalition, Duivendrecht‐Amsterdam Duivendrecht The Netherlands; ^6^ Genetics and Genome Biology The Hospital for Sick Children Toronto Ontario Canada; ^7^ Department of Medical Genetics University of Cambridge Cambridge UK; ^8^ NIHR Cambridge Biomedical Research Centre Cambridge Biomedical Campus Cambridge UK; ^9^ Division of Medical Oncology and Hematology, Princess Margaret Cancer Centre University Health Network and Sinai Health System Toronto Ontario Canada; ^10^ Division of Clinical and Metabolic Genetics The Hospital for Sick Children Toronto Ontario Canada; ^11^ Ontario Institute for Cancer Research Toronto Ontario Canada; ^12^ Department of Medicine University of Toronto Toronto Ontario Canada

**Keywords:** CIViC, genotype–phenotype, machine learning, spectral clustering, Von Hippel‐Lindau

## Abstract

Von Hippel‐Lindau (VHL) disease is a hereditary cancer syndrome where individuals are predisposed to tumor development in the brain, adrenal gland, kidney, and other organs. It is caused by pathogenic variants in the *VHL* tumor suppressor gene. Standardized disease information has been difficult to collect due to the rarity and diversity of VHL patients. Over 4100 unique articles published until October 2019 were screened for germline genotype–phenotype data. Patient data were translated into standardized descriptions using Human Genome Variation Society gene variant nomenclature and Human Phenotype Ontology terms and has been manually curated into an open‐access knowledgebase called Clinical Interpretation of Variants in Cancer. In total, 634 unique *VHL* variants, 2882 patients, and 1991 families from 427 papers were captured. We identified relationship trends between phenotype and genotype data using classic statistical methods and spectral clustering unsupervised learning. Our analyses reveal earlier onset of pheochromocytoma/paraganglioma and retinal angiomas, phenotype co‐occurrences and genotype–phenotype correlations including hotspots. It confirms existing VHL associations and can be used to identify new patterns and associations in VHL disease. Our database serves as an aggregate knowledge translation tool to facilitate sharing information about the pathogenicity of *VHL* variants.

## 1 INTRODUCTION

Von Hippel‐Lindau (VHL) disease is a rare hereditary cancer predisposition syndrome with an estimated prevalence of 1 in 31,000–53,000 (Maher et al., 2011). Affected individuals are at risk of cyst and tumor development in multiple organs, including the central nervous system (CNS) and renal, pancreatic, adrenal, and reproductive organs (Maher, 1994). These individuals inherit an autosomal dominant pathogenic inactivating variant in the *VHL* gene (Maher, 1994), although approximately 20% of cases have been suggested to arise de novo (F. M. Richards et al., 1995). The *VHL* gene encodes for the VHL protein (pVHL), a critical regulator of the hypoxia‐inducible transcription factor α (HIF‐α) (Maxwell et al., 1999). In normoxia, the β‐domain of pVHL binds to HIF‐α while the α‐domain of pVHL binds to the E3 ubiquitin ligase complex that targets HIF‐α for proteasomal degradation (Maxwell et al., 1999). In VHL disease, tumorigenesis is dependent on a pathogenic germline variant (“first‐hit”) and the subsequent somatic inactivation of the remaining wildtype allele (“second‐hit”) (Knudson, 1971; Prowse et al., 1997). Under normal conditions, pVHL is ubiquitously expressed in normal human tissue (F. M. Richards et al., 1996); in tissues with two “hits,” the deficiency in pVHL results in the overexpression of HIF‐α, leading to unregulated angiogenesis and highly vascularized tumor development (Knudson, 1971; Maxwell et al., 1999).

The most common manifestations of VHL are retinal angioma (RA), CNS hemangioblastoma (CHB), pheochromocytoma/paraganglioma (PPGL), renal cell carcinoma (RCC) and renal cysts, pancreatic neuroendocrine tumor (PNET) and pancreatic cysts, endolymphatic sac tumors (ELST), and epididymal and broad ligament cysts (Maher, 1994). Diagnosing VHL disease can be complex; currently there are several diagnostic criteria guidelines, such as the Danish, Dutch, and International guidelines (Choyke et al., 1995; Hes et al., 2005; Hes & van der Luijt, 2000; Lonser et al., 2003; Maher et al., 2011; Nordstrom‐O'Brien et al., 2010; VHL Alliance, 2010). Despite subtle nuances, all guidelines agree that a family history for VHL and one manifestation or two VHL manifestations in the absence of a family history should raise a clinician's suspicion of VHL disease (Choyke et al., 1995; Hes et al., 2005; Hes & van der Luijt, 2000; Lonser et al., 2003; Maher et al., 2011; Nordstrom‐O'Brien et al., 2010; VHL Alliance, 2010). For example, the Danish criteria suggest that a clinical diagnosis of VHL disease is made if a suspected patient has (1) two VHL‐associated tumors, (2) one VHL‐associated tumor and a pathogenic variant in the *VHL* gene, or (3) one VHL‐associated tumor and at least one first‐degree relative with VHL disease (Binderup et al., 2013). Symptoms generally develop in early adulthood (average age of diagnosis ranges widely from 20 to 40 years) (Maher, 1994; Maher et al., 2011), although reports have also described symptomatic pediatric patients under 5 years of age (Aronoff et al., 2018; Sovinz et al., 2010). Identification of a pathogenic *VHL* germline variant is also important for identifying asymptomatic relatives who may be at risk of tumorigenesis later in life (Maher & Kaelin, 1997; Nordstrom‐O'Brien et al., 2010). The highest mortality rates are caused by complications from RCC and CHBs (Binderup et al., 2017); to reduce VHL‐associated morbidity, minimum surveillance guidelines have been recommended for all carriers of pathogenic *VHL* germline variants. The 2020 VHL Alliance Surveillance Guidelines suggest starting in childhood and include annual physicals, dilated eye exams, metanephrines, biennial abdominal imaging, and craniospinal magnetic resonance imaging (VHL Alliance, 2010).

Early correlation studies have classified VHL disease into VHL type 1, associated with truncating (e.g., frameshift, deletion, nonsense) variants and the absence of PPGL and VHL type 2, associated with missense variants and PPGL (Chen et al., 1996; Hes et al., 2000; Nielsen et al., 2016; Ong et al., 2007). More specific genotype–phenotype correlations can help stratify patients by their risk of developing certain phenotypes and further personalize their care. For example, a study in Korean families found a decreased risk of CHB, RCC, and PNET in patients with *VHL* missense variants outside the HIF‐α binding site, compared with patients with missense *VHL* variants in the HIF‐α binding site and truncating variants (Liu et al., 2018). Missense mutations were previously reported in association with PNETs in VHL disease (Blansfield et al., 2007); this was further reported by an association between missense mutations and larger PNET diameter, as well as the development of metastatic disease and requirement for surgical intervention (Tirosh et al., 2018). More recently, a retrospective study of 577 VHL cases in a Chinese population identified more specific genotype–phenotype correlations by classifying patients into groups based on their variant type and location (Qiu et al., 2020). Patients with nonsense or frameshift variants occurring upstream of codon 117 had a lower age‐related risk of VHL‐associated tumors and longer median lifespan than patients with nonsense or frameshift variants downstream codon 117 (where the HIF‐α binding site occurs) (Qiu et al., 2020). Patients with missense pathogenic variants occurring outside of functional domains in the *VHL* gene had a higher risk of pheochromocytoma; this was protective against the development of CHB (Qiu et al., 2020). Missense variants in the HIF‐α binding site were also found to be associated with age‐related risk of CHB (Lee et al., 2016). Critical amino acids can also be identified through recurrent variant observations and variants that share the same codon as previously observed variants are under increased suspicion for pathogenicity (S. Richards et al., 2015). Two decades ago, six recurrently altered amino acids, or hotspots, in the *VHL* gene (NM_000551.3) were described (codons 78, 86, 96, 162, 167, 178) (Stebbins et al., 1999) and a large study on 540 Chinese patients confirmed codon 167 as a hotspot and also identified codons 65 and 161 (Hong et al., 2019).

While these past studies are extremely valuable, they provide isolated pieces of information and often draw from homogenous and/or familial populations. As a result, clinical surveillance guidelines for VHL patients often rely on clinical judgment and do not distinguish between VHL subtypes. Variability in clinical manifestation between patients, including age of presentation and which organs are involved in tumorigenesis, makes it difficult to develop and optimize standard international guidelines (Kruizinga et al., 2014; Poulsen et al., 2010). Furthermore, due to the rarity of VHL disease and the use of gene panel testing in clinical practice, variants of uncertain significance (VUS) are regularly encountered (Hoffman‐Andrews, 2017), with no singular method to aid in determining their pathogenicity. Current strategies involve a clinical evaluation of all cases reported in the literature and genetic, histology, and immunohistochemistry analyses of tumor tissue, as well as assessment of the variants' impact on the protein structure (Alosi et al., 2017). This process requires extensive time and resources.

Thus far, there have been limited efforts to aggregate genotype–phenotype information for VHL patients on a meta‐analysis scale. Databases such as VHLdb (Tabaro et al., 2016) are thorough, but lack an interface for analyzing interpreted and standardized *VHL* variants and their associated phenotypes. Takayanagi et al. (2017) have also described a database on a large population of Japanese VHL patients, but conclusions drawn from this sample may not be generalizable to other populations. Due to the novelty of VHL disease research, new reports are regularly published; however, there is currently no systematically updated database that thoroughly standardizes all VHL patient and variant information reported in the literature. To address these gaps, we have curated the largest standardized open‐access database on VHL patient genotype–phenotype data using the Clinical Interpretation of Variants in Cancer (The McDonnell Genome Institute (2022); https://civicdb.org/) knowledgebase (Griffith et al., 2017). CIViC is an expert‐crowdsourced knowledgebase supporting up‐to‐date variant interpretations and open‐access content (Griffith et al., 2017). CIViC data can be freely accessed as evidence records, where each record describes one unique *VHL* variant from a publication and includes information such as associated disease, associated Human Phenotype Ontology (HPO) terms and a freeform text “evidence statement.” This “evidence statement” includes a clinical description of families and patients associated with the variant in the publication source. The variant identity (often reported ambiguously in the primary literature) is resolved to unambiguous genomic coordinates and linked to the Clinical Genome Resource (ClinGen) Allele Registry (Huang et al., 2017) and other variant interpretation databases and algorithmic predictions of functional impact. The data in CIViC undergoes an iterative editing and verification process by trained CIViC editors; all data are easily accessible by public users for live comments, discussion, and engagement.

This standardization and aggregation of VHL patient information also facilitates higher‐level analysis, where overarching trends can be identified from the larger body of data. The use of machine learning techniques in modern healthcare is becoming ubiquitous and such methods can incorporate many facets of patient genetic information (Huang et al., 2017). Unsupervised machine learning techniques, such as spectral clustering, use underlying patterns in the data rather than requiring known constraints, and labeling as inputs (Huang et al., 2017). Such methods have been successfully implemented in precision medicine and are used extensively in survival analysis (Huang et al., 2017). A strength of this method is the ability to incorporate a variety of patient and genetic data, such as sequence variants, phenotypes, and gene expression information, to elucidate patterns and reveal insights that aid in the treatment of an individual (Huang et al., 2017). Herein, we report on the analyses of the largest aggregate VHL patient population to date, consisting of all published VHL cases with relevant genotype–phenotype information. Using spectral clustering, we identified relationship trends between VHL patient phenotype and genotype in our aggregated data. Our large‐scale analyses confirm previously reported genotype–phenotype correlations and reveal novel findings to deepen our understanding of VHL disease.

## 2 METHODS

### 2.1 Literature search and VHL data set curation

**Figure 1:**
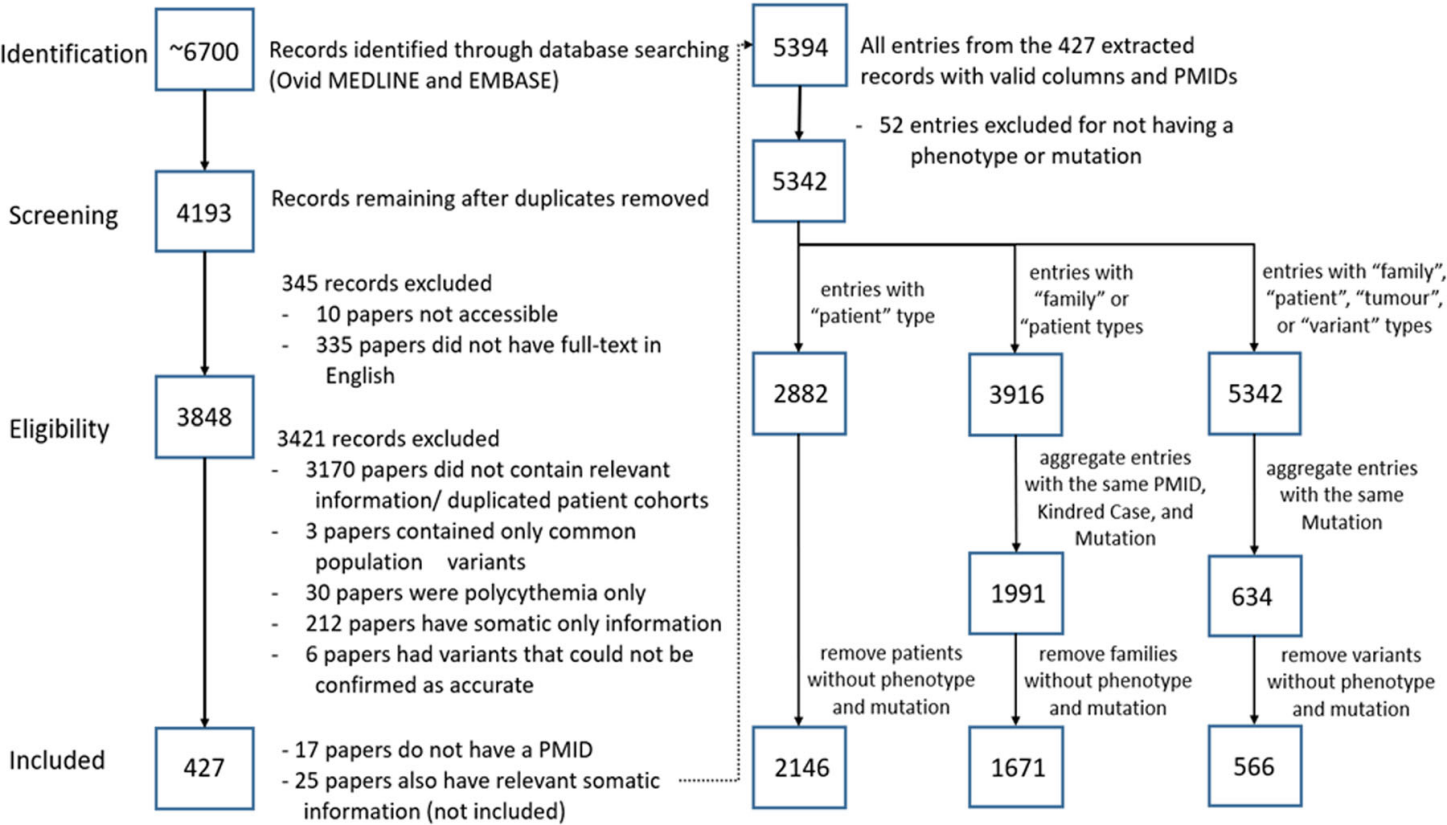
Number of VHL papers analyzed at each state of the identification, screening, eligibility, and inclusion process. Four hundred and twenty‐seven papers were included in the final analysis assessing VHL genotype–phenotype correlations. VHL, Von Hippel‐Lindau.

A basic literature search was conducted on September 30, 2019. Key concepts “(Von Hippel‐Lindau) AND (genetics OR gene mutations)” and “(Von Hippel‐Lindau) and (databases)” were searched in the Ovid MEDLINE and Ovid EMBASE databases. Key concepts were searched for in the title, abstract, and index terms. Articles published until October 2019 (including preprint articles) were retrieved for full text review and were included in the current study based on the following criteria: (1) paper described and reported germline *VHL* variants and (2) the variants were found in patients fulfilling the clinical criteria for VHL disease (Binderup et al., 2013) and/or were associated with the VHL phenotypes previously described (Maher, 1994; Maher et al., 2011). Literature reviews, non‐English papers, and papers only describing VHL patients without phenotypic data were excluded (Figure 1).

To validate the relevance of our database, the articles in the search were compared against two known publicly available VHL databases: VHLdb (Tabaro et al., 2016) and the Universal Mutation Database‐VHL (UMD‐VHL; Barlier & Mohamed, 2021) (Béroud et al., 2000). VHLdb includes 78 publications containing germline information from 1990 to 2018, and UMD‐VHL includes 91 publications up to May 25, 2021. These publications were compared to all papers from our literature search and papers which fulfilled our database inclusion criteria.

#### 2.1.1 Data set study population

While some papers report individual data points, others present data in summarized forms, which can be a challenge when integrating individual data sets (Ritchie et al., 2015). To account for different data types, quality assessment, each case was classified into four categories: (1) individual‐level data (“Patient” resolution), (2) data aggregated by family (“Family” resolution), (3) data aggregated by variant (“Variant” resolution), and (4) tumor studies and/or papers where data on only one tumor type was available (“Tumor” resolution) (Supporting Information: Table S1). Patients reported in the same paper who belong to the same family are identified by a unique kindred ID. If a paper explicitly identified that a VHL case was previously published, the publications were compared and only one VHL case with the most complete information was extracted to address duplicated data. Where appropriate, the source record in CIViC corresponding to such publications were annotated to describe the nature and extent of overlapping cases between studies to improve the efficiency of *VHL* variant curation efforts.

For our analysis, we drew from the VHL cases captured in the database with both genotype and VHL‐associated phenotype information. Because entries with no phenotype and mutation type data could not provide meaningful information, entries without both were filtered out. Patient‐based analysis only included those cases tagged with “Patient” resolution. Family‐based analysis included cases tagged with “Family” resolution and cases tagged with “Patient” resolution, where related “Patient” cases were aggregated into families using the unique kindred ID. One data point in the family‐based analysis represents one family and all associated phenotypes, where at least one member was genetically confirmed with a *VHL* variant. The variant‐based analysis included all four data types (“Patient,” “Family,” “Tumor,” and “Variant”), where one data point represents one unique *VHL* variant with all associated phenotypes. In all types of analysis, data points were filtered again after aggregation to ensure they had both genotype and VHL‐associated phenotype information.

#### 2.1.2 Genotype data

All published *VHL* variants were standardized according to Human Genome Variation Society (HGVS)‐nomenclature (den Dunnen et al., 2016) and *VHL* reference sequence NM_000551.3. Variant descriptions were validated with VariantValidator.org (University of Manchester & University of Leicester, 2022; Freeman et al., 2018) and any inconsistencies were reviewed by an expert team of genetic counselors, geneticists, genomic researchers, and CIViC knowledgebase editors. Reported variants that could not be verified, either by the expert team or the publication authors, were excluded from the study. Only VHL cases with genetically confirmed variants were recorded, however, due to the heritability pattern of VHL disease, family members without genetic testing available but with genetically confirmed relatives were also curated with an “unknown” genotype in the database. Cases were confirmed de novo only if supported by both maternal and paternal genetic testing.

The Genome Aggregation Database (Rehm & Daly, n.d.) v2.1.1 utilizes whole exome and whole genome sequencing data from 141,456 unrelated individuals from diverse populations with extensive variant calling and filtering to report germline variant observations in these samples. The gnomAD database excludes severe pediatric cases and their first‐degree relatives, allowing gnomAD to serve as a control data set to largely represent the general population (Karczewski et al., 2019). Extracted *VHL* variants were identified as common population variants, unlikely to contribute to VHL disease, if they were described on gnomAD with a global minor allele frequency >1% (Kruglyak & Nickerson, 2001). These cases were excluded from the analysis and summarized separately from the rest of the data set (Supporting Information: Table S2). For patients with heterozygous compound variants, where one variant was common, the common population variant was excluded from analysis and the patient was considered heterozygous for *VHL* variants.

For the analysis on the aggregated data, family members without genetic confirmation but who fulfilled a clinical diagnosis of VHL disease were assumed to carry the same familial *VHL* variant—consistent with VHL diagnosis guidelines (Binderup et al., 2013). This assumption was only used for our analysis and was not carried over to the datasheet; therefore, future database users will have a record of genetically confirmed cases.

#### 2.1.3 Phenotype data

Reported phenotypes were extracted as HPO terms, which are the standard phenotype annotations for many large‐scale rare disease genome databases (Köhler et al., 2019). HPO terms can be used for computational deep phenotyping, whereby phenotypes are related to more specialized (child) and less specialized (parent) phenotypes in directed acyclic graphs (DAG) (Köhler et al., 2019). The application of DAG is especially relevant when aggregating heterogeneous data from different sources (e.g., “brain tumor” vs. “CHB”). Reported phenotypes were annotated with the corresponding HPO definitions with the exception of two cases: (1) the parent term “hemangioblastoma” was only used for CHB cases and not RA and (2) the parent term “neuroendocrine neoplasm” was used for cases of PPGL and not PNET.

To be consistent with past and current descriptions of VHL disease and to maximize compatibility of heterozygous data, we combined the annotated HPO terms for our analysis. We used the following phenotypes: “CHB,” “RA,” “PPGL,” “RCC,” “PNET,” “ELST,” “pancreatic cysts or tumors (PCT),” “renal cysts or tumors (RCT),” “epididymal cysts or tumors,” and “ovarian cysts or tumors (OCT).” For example, the phenotype CHB consisted of three HPO terms: spinal hemangioblastoma (HP:0009713), cerebellar hemangioblastoma (HP:0006880), and hemangioblastoma (HP:0010797). A breakdown of all phenotypes used in our analysis is available through the Supporting Information: Material and Methods (Supporting Information: Table S1).

#### 2.1.4 Age and sex

Information on age and sex was extracted when available. Pairwise deletion was used to address missing data. To address the differences in how age was reported between studies, we categorized age into three types: (1) age of onset—defined as the reported age of the patient when they first presented with a VHL‐associated phenotype, (2) age of death, and (3) last known age of the patient.

#### 2.1.5 CIViC knowledgebase curation

The usability and features of CIViC have previously been described (Griffith et al., 2017). VHL cases were curated into structured evidence records and summarized with “evidence statements” using the CIViC evidence curation interface. To incorporate our standardizations in *VHL* variant curation, reviewers were trained on the American College of Medical Genetics and Genomics (ACMG) guidelines (S. Richards et al., 2015) with assigned ACMG evidence criteria incorporated into the “evidence statement.”

### 2.2 Statistical analysis

#### 2.2.1 Summary characteristics of study population

Demographic information of patients, including the frequency of patients with de novo variants, the ratio of male to female patients, mean age of onset, last known age, and age of death were calculated across the data where pertinent information for the patient was present. Family‐, tumor‐, and variant‐based data did not have distinguishable demographic information. The frequency of different phenotypes and variant types was calculated for patient‐, family‐, and variant‐based data.

#### 2.2.2 Age‐related penetrance of phenotypes

Due to inconsistent reporting, the current data set does not have access to patient age at the onset of each new phenotype presentation. To model age‐related penetrance of phenotypes, we limited our sample to patients with only one phenotype to extrapolate age of onset and phenotype(s).

#### 2.2.3 Phenotype co‐occurrence ratio

The correlations between two phenotypes occurring together in the same patient, family and variant were calculated as co‐occurrence ratios. This ratio considers overall pairwise likelihoods between phenotypes.

#### 2.2.4 High‐frequency missense variants

Patient‐ and family‐based data were used to assess for high‐frequency missense variants in the VHL population. Variant‐based data were used to assess the frequency of unique variants occurring in a codon. A one‐tailed binomial test was used to determine if any of the 213 codons had variant counts statistically greater than if the variants were distributed evenly. Secondary analysis was also performed by first adjusting the codon counts via Blocks Substitution Matrix (BLOSUM)90 and then using a one‐tailed binomial test on these codon counts (Pearson, 2013; Tavtigian et al., 2008; Vitkup et al., 2003). A test was run for each codon and the α level of significance was adjusted via the Bonferroni correction, which decreases the risk of type I errors associated with multiple statistical tests (Armstrong, 2014).

#### 2.2.5 Phenotype–genotype correlations

Variants were categorized into truncating (stop‐gained, frameshift, deletions, splice) and nontruncating variants (missense, inframe‐deletions, inframe‐insertions). Synonymous, untranslated region (UTR) and intronic regions were not included in these groupings. The ratio of truncating and nontruncating variants for each phenotype were assessed for patient‐, family‐, and variant‐based data using a contingency table with *χ* ^2^ post hoc analysis. A post hoc test was run for each pair of phenotypes and the α level of significance was adjusted via the Bonferroni correction (Armstrong, 2014).

Coding variants were identified as occurring in structural and functional domains. The α‐domain was defined as codons 156–204 and β‐domain codons 63–143, inclusive (Min et al., 2002). The HIF‐α binding site and the Elongin B and C binding site were defined as codons 67, 69, 75, 77–79, 88, 91, 98–99, 105–112, 115, 117 and 79, 153, 159, 161–163, 165–166, 174, 177–178, 184, respectively (Min et al., 2002). An 8 × 5 amino acid tandem repeat region, labeled (GXEEX)8 and defined as codons 14–53, was also included in the analysis. The frequency of both truncating and nontruncating variants occurring in each domain and region was assessed for patient‐, family‐, and variant‐based data. A *χ* ^2^ test was used to find differences in phenotypes' distributions of variants across the structural and functional domains in the *VHL* gene.

#### 2.2.6 Machine learning: Cluster analysis

To confirm the presence of known VHL type 1 and type 2 classifications in our data set, we implemented spectral clustering, an unsupervised learning algorithm (Ng et al., 2001). Spectral clustering was chosen as the unsupervised learning method because it groups patients without knowing their diagnoses or VHL type classifications and has been used successfully in the discovery of genotype–phenotype correlations (Pai & Bader, 2018; Wang et al., 2014). The network was constructed with each node representing a single patient and the edge between any two patients representing the similarity of their VHL phenotype manifestations. The score used to calculate phenotype similarities between patients is the Jaccard similarity score, where patients with higher scores have the most similar overlapping phenotypes (Jaccard, 1912). Patients that did not have any VHL‐related phenotypes were omitted from cluster analysis. To automatically determine the optimal number of clusters present in the data, the eigengap method was used (von Luxburg, 2007). The two largest gaps in the Laplacian matrices' eigenvalues were used, producing two estimates for the number of groups of patients. Therefore, clustering was run twice and the results for both group estimates were analyzed. To draw parallels to known VHL type classifications, genotypic statistics were calculated for each group of patients and compared to one another.

#### 2.2.7 Penetrance

A two‐sample Kolmogorov–Smirnov test with a 95% confidence interval was performed for each pair of distributions of isolated phenotypes in patients, and the Bonferroni correction was used to adjust the α threshold of significance (Armstrong, 2014). The null hypothesis is that there is no difference between the two phenotype distributions and the alternative hypothesis would be there are any differences.

## 3 RESULTS

### 3.1 Complete VHL datasheet and CIViC knowledgebase

In all, 634 unique *VHL* variants, 2882 patients and 1991 families from 427 papers were captured in our data set. The raw data, including information on genotype, phenotype and sample characteristics, can be accessed in an expanded datasheet (Supporting Information: Table S1). Centralized and interpreted data on *VHL* variants is publicly available on CIViC (https://civicdb.org/). As the CIViC knowledgebase requires a PubMed ID (PMID) for each entry, 17 papers had data used in our analysis but did not have accessible reference numbers to be uploaded online.

There were 53 papers that were included in UMD‐VHL and/or VHLdb and not in our data set, of which 49 were captured in our literature search. These papers were excluded for not being in English, not having germline *VHL* variants and/or no relevant data on VHL patients or phenotypes. Four papers not captured in our literature search were reviewed and determined not to fulfill the inclusion–exclusion criteria. Three hundred and sixty‐one papers were captured in our data set that were not found on either UMD‐VHL or VHLdb (including 17 papers without a PMID).

### 3.2 Summary characteristics of study sample

Characteristics of the study population are summarized in Table 1. We analyzed data from 2146 patients, 1671 families and 566 variants with relevant genotype–phenotype information. Of the 1302 patients with gender annotated, 682 (52.4%) were male. The patient's last known age was available for 1460 patients, with a mean age of 33.4 ± 15.4 years (range = 0–86 years, median = 32 years). The age of onset, defined as the age of any VHL disease manifestation, was available for 1031 patients, with a mean age of 27.7 years (range = 0–79 years). De novo variants were confirmed in 72 patients. Finally, CHB and missense variants were found to be the most frequent VHL manifestation (58.0%) and variant type (62.4%), respectively.

**Table 1:** Sample characteristics for VHL patients, families, and variants.

	Patient (*N* = 2146)	Family (*N* = 1671)	Variant (*N* = 566)
Total *N* with sex data	*N* = 1302	N/A	N/A
Male	682 (52.4%)		
Female	620 (47.6%)		
Total *N* with age data	*N* = 1460	N/A	N/A
Last known age mean (*N* = 1461, range = 0–86 years)	33.4 years		
Age of onset mean (*N* = 1030, range = 0–79 years)	27.7 years		
*N* with confirmed de novo	*N* = 72	N/A	N/A
Phenotype distribution	*N* = 2146	*N* = 1671	*N* = 566
PNET	152 (7.1%)	138 (8.3%)	111 (19.6%)
PPGL	797 (37.1%)	618 (37.0%)	233 (41.2%)
CHB	1248 (58.2%)	1167 (69.8%)	389 (68.7%)
RA	748 (34.9%)	771 (46.1%)	328 (58.0%)
RCC	675 (31.5%)	710 (42.5%)	292 (51.6%)
RCT	321 (15.0%)	336 (20.1%)	174 (30.7%)
PCT	583 (27.2%)	573 (34.3%)	218 (38.5%)
Variant type distribution	*N* = 2146	*N* = 1671	*N* = 566
Missense variant	1339 (62.4%)	931 (55.7%)	274 (48.4%)
Exon loss variant	157 (7.3%)	182 (10.9%)	30 (5.3%)
Deletion	167 (7.8%)	181 (10.8%)	47 (8.3%)
Stop gained	171 (8.0%)	149 (8.9%)	37 (6.5%)
Splice site variant	82 (3.8%)	59 (3.5%)	32 (5.7%)
Frameshift variant	168 (7.8%)	149 (8.9%)	148 (26.1%)
Intron variant	1 (0.0%)	4 (0.2%)	5 (0.9%)
Synonymous variant	31 (1.4%)	16 (1.0%)	12 (2.1%)
Inframe indel	42 (2.0%)	44 (2.6%)	26 (4.6%)
Stop lost	6 (0.3%)	5 (0.3%)	5 (0.9%)
Start lost	1 (0.0%)	1 (0.1%)	1 (0.2%)
Delins	7 (0.3%)	9 (0.5%)	12 (2.1%)
UTR region variant	12 (0.6%)	10 (0.6%)	8 (1.4%)
	8 (0.4%)		
	13 (0.6%)		
Variant grouping			
Truncating	*N* = 647	*N* = 615	*N* = 233
PNET	31 (4.8%)	34 (5.5%)	36 (15.5%)
PPGL	72 (11.1%)	90 (14.6%)	63 (27.0%)
CHB	519 (80.2%)	563 (91.5%)	197 (84.5%)
RA	250 (38.6%)	319 (51.9%)	142 (60.9%)
RCC	269 (41.6%)	334 (54.3%)	152 (65.2%)
RCT	134 (20.7%)	153 (24.9%)	83 (35.6%)
PCT	269 (41.6%)	169 (27.5%)	104 (44.6%)
Nontruncating	*N* = 1374	*N* = 966	*N* = 277
PNET	112 (8.2%)	98(10.1%)	66 (23.8%)
PPGL	688 (50.1%)	511 (52.9%)	158 (57.0%)
CHB	676 (49.2%)	587 (60.8%)	177 (63.9%)
RA	466 (33.9%)	433 (44.8%)	168 (60.6%)
RCC	363 (26.4%)	348 (36.0%)	125 (45.1%)
RCT	167 (12.2%)	161 (16.7%)	84 (30.3%)
PCT	277 (20.2%)	259 (26.8%)	105 (37.9%)

*Note*: Age, phenotype, variant type, and confirmed de novo statistics are shown for each of the patient, family, and variant analysis. The statistics for variant types grouped into truncating or nontruncating mutations are also shown for each phenotype and analysis type.

Abbreviations: CHB, CNS hemangioblastoma; CNS, central nervous system; PCT, pancreatic cysts or tumors; PNET, pancreatic neuroendocrine tumor; PPGL, pheochromocytoma/paraganglioma; RA, retinal angioma; RCC, renal cell carcinoma; RCT, renal cysts or tumors; VHL, Von Hippel‐Lindau.

### 3.3 Penetrance

**Figure 2:**
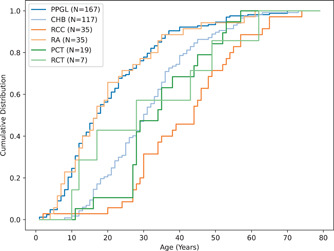
Age‐related penetrance for patients that present with a single phenotype. PPGL and RA have an earlier age of onset compared to the other phenotypes (RCT, CHB, RCC, and PCT). CHB, CNS hemangioblastoma; CNS, central nervous system; PCT, pancreatic cysts or tumors; PPGL, pheochromocytoma/paraganglioma; RA, retinal angioma; RCC, renal cell carcinoma; RCT, renal cysts or tumors.

After removing patients who did not have an isolated phenotype, too few patients remained to plot the age distribution for PNET's. The remaining six phenotypes (PCT, RCT, PPGL, RA, CHB, RCC) were compared pairwise, resulting in $\frac{6\left(6-1\right)}{2}=15$ comparisons (Supporting Information: Table S3). Using the Bonferroni correction, we set the threshold of significance to be $\alpha =\frac{0.05}{15}=0.0033$. There were no significant statistical differences between RCT and any other phenotype distribution (p>0.0033). The distributions for age of onset for PPGL and RA were not statistically different from each other (p>0.0033), but were both statistically different than CHB, RCC, and PCT (p<0.0033). The age distribution for RCC was significantly different from CHB, but not PCT or RCT. Combined with the information in Figure 2, this suggests that PPGL and RA had an earlier age of penetrance than CHB, RCC, and PCT, with 50% of those patients presenting PPGL and RA before the age of 18. For patients with CHB, RCC, and PCT, the 50% patient threshold was 36, 44, and 27 years of age, respectively. Additionally, RCC had an age of onset later than CHB, PCT, and RCT.

### 3.4 Phenotype analysis: Co‐occurrence

**Figure 3:**
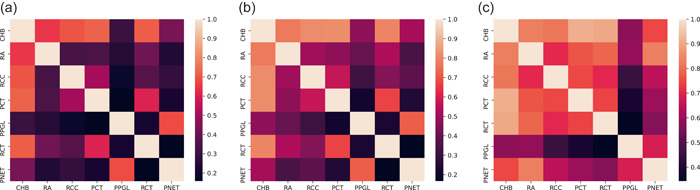
Phenotype co‐occurrence ratios for (a) patient‐, (b) family‐, and (c) variant‐based data. In all instances, PPGL had a low co‐occurrence ratio for all data‐types, except PNET. CHB had a high co‐occurrence ratio with all phenotypes except PPGL and PNET in patient‐ and family‐based data; a low co‐occurrence ratio was seen between CHB and PPGL, moderate co‐occurrence between CHB and PNET, and high co‐occurrence of CHB with all other phenotypes. CHB, CNS hemangioblastoma; CNS, central nervous system; PNET, pancreatic neuroendocrine tumor; PPGL, pheochromocytoma/paraganglioma.

The co‐occurrence phenotype ratios for patient, family, and variant data are visualized in Figure 3a–c, respectively. Ratios were scaled between 0 and 1 per manifestation. We defined low co‐occurrence as 0–0.33, moderate as 0.34–0.66, and high as 0.67–1. Notably, across all data‐types, PPGL had a low co‐occurrence ratio with all phenotypes except PNET. CHB had a high co‐occurrence ratio with all phenotypes except PPGL and PNET in patient‐ and family‐based data. In variant‐based data, CHB had a low co‐occurrence ratio with PPGL, moderate co‐occurrence with PNET and high co‐occurrence with all other phenotypes.

### 3.5 Variant analysis: Hotspot missense variants

**Figure 4:**
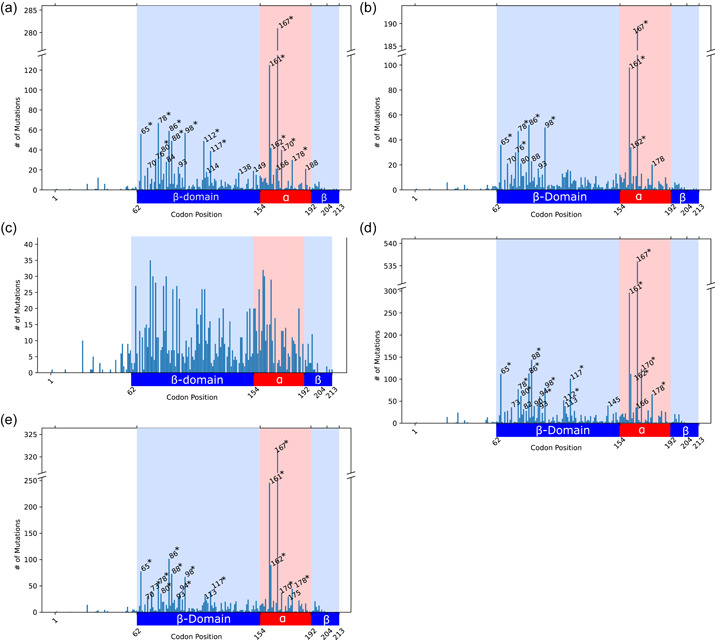
Frequency of missense variants along the *VHL* gene for (a) patient‐, (b) family‐, and (c) variant‐based data, and BLOSUM90‐adjusted missense frequency for (d) patient‐ and (e) family‐based data. Codons identified as hotspots are labeled with numbers and asterisks (*) indicate highly significant hotspots. The α‐domain is indicated by the red background region and the β‐domain is indicated by the blue background region.

High‐frequency missense variants in the VHL population were identified in patients and families. The one‐tailed binomial tests (Supporting Information: Table S4) showed that codons with ≥21 or ≥17 missense variants for patients and families, respectively, were below the confidence interval of $\alpha =0.05\;(\text{adjusted to}\;\frac{0.05}{213}=0.000235)$ and the null hypothesis that the variants were distributed evenly was rejected for these codons. Figure 4a illustrates all the statistically high frequency codons for patients. Codons with >30 missense variants which had a p value four orders of magnitude below the adjusted α interval are indicated by an asterisk (*). Figure 4b shows all of the high frequency codons for families with asterisks indicating highly significant hotspots. Variant‐based analysis showed no notable codon locations with a high‐frequency of unique variants (Figure 4c). This analysis was repeated after adjusting the codon counts based on underlying amino acid conservation rates captured by the BLOSUM90 matrix. While the binomial test and the confidence interval remained the same for this analysis, rejection of the null hypothesis now meant that the BLOSUM‐adjusted scores were not evenly distributed across the codons. This was the case for codons with cumulative BLOSUM scores of ≥32 and ≥28.6 for missense variants for patients and families, respectively. Figure 4d,e shows the high BLOSUM‐adjusted frequency codons for patients and families.

### 3.6 Phenotype–genotype correlations

**Figure 5:**
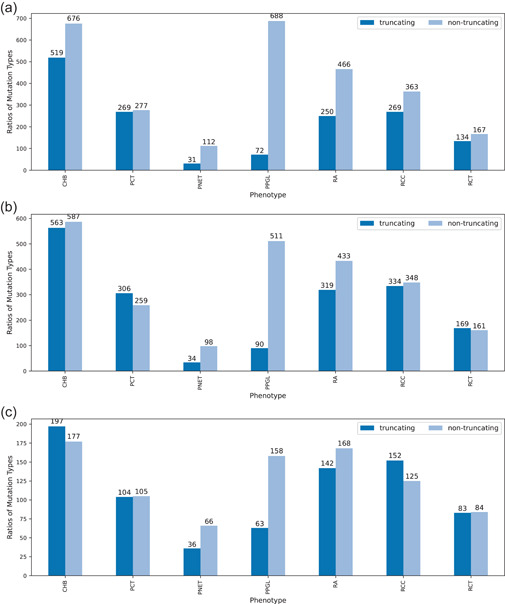
Distribution of truncating and nontruncating variant by phenotype for (a) patient‐, (b) family‐, and (c) variant‐based data. Significant differences were seen between truncating/nontruncating distributions between phenotypes. In particular, nontruncating variants were favored over truncating variants for PPGL; results of PNETs distribution was also statistically different from all other phenotypes except RA, to favor nontruncating variants. PNET, pancreatic neuroendocrine tumor; PPGL, pheochromocytoma/paraganglioma; RA, retinal angioma.

The distribution of truncating and nontruncating variants by phenotype for patient‐, family‐, and variant‐based data are summarized in Figure 5a–c, respectively. The *χ* ^2^ statistical test (Supporting Information: Table S5) showed that there was a significant difference between truncating/nontruncating distributions between phenotypes ($\chi^{2}=333,p=5.28\times 10^{-69}$). Pairwise post hoc *χ* ^2^ tests (with corrected $\alpha =\frac{0.05}{21}=0.00238$) showed that PPGL's ratio of nontruncating to truncating variants was statistically different than all other phenotype distributions, heavily favoring nontruncating variants. Similarly, PNET's distribution was statistically different from all other phenotypes except RA, favoring nontruncating over truncating variants. RA also favored nontruncating over truncating variants, but was not statistically different from RCC. Of patients with PPGL and PNET, 90% and 79% harbored nontruncating variants, respectively. In families with PPGL and PNET, 85% and 75% had nontruncating variants and 72% and 63% of unique variants associated with PPGL and PNET, respectively, were nontruncating.

**Figure 6:**
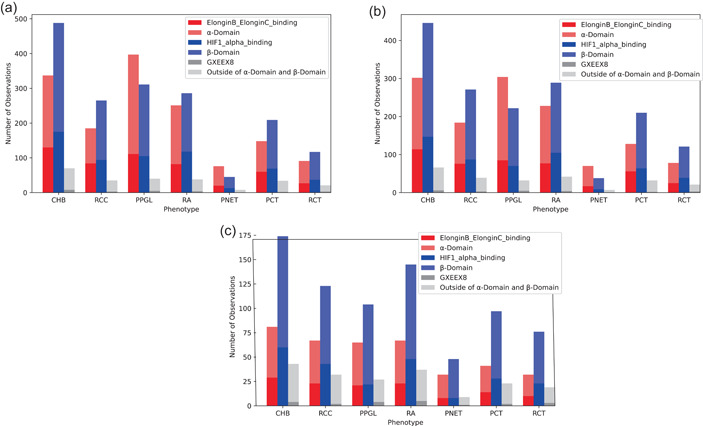
Frequency of coding variants in protein and functional domains for (a) patient‐, (b) family‐, and (c) variant‐based data. PPGL and PNET were statistically different (*p *< 0.00238) than all other phenotypes, but not each other and favored variants being distributed in the α‐domain over the β‐domain. PNET, pancreatic neuroendocrine tumor; PPGL, pheochromocytoma/paraganglioma.

The frequency of variants occurring in each phenotype, broken down by structural and functional domains, is summarized in Figure 6. The *χ* ^2^ statistical test (Supporting Information: Table S6) showed that there was a significant difference between α‐domain and β‐domain distributions across the phenotypes $\left(\chi^{2}=59.3,p=6.21\times 10^{\;-\;11}\right)$. PPGL and PNET were statistically different (p<0.00238) than all other phenotypes, but not each other and favored variants being distributed in the α‐domain over the β‐domain. There were no significant differences in functional domain distributions between any other phenotype pairs (Figure 6a,b). Although both PPGL and PNET phenotypes had more variants in the Elongin B and C binding site than the HIF‐α binding site, none of the differences in functional site distributions were found to be significant $\left(\chi^{2}=7.95,p>0.00238\right)$ (Supporting Information: Table S7).

### 3.7 Machine learning: Cluster analysis

For patient‐based clustering, it was determined (via the eigengap estimation) that the best estimates for the number of patient clusters were *K* = 2 and *K* = 4. As our spectral clustering metric was based solely on phenotype similarity between patients, the algorithm separated the patient clusters by phenotypes.

**Figure 7:**
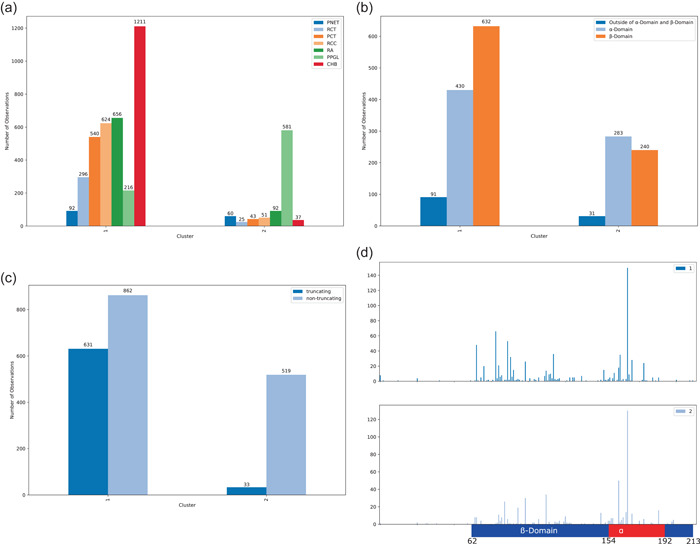
Cluster phenotype, variant type, variant domain and codon distribution for two patient clusters. Patient cluster 2 was PPGL dominant (a) and had slightly more variants in the α‐domain, whereas cluster 1 had more variants in the β‐domain (b). Cluster 2 heavily favored nontruncating variants over truncating, whereas cluster 1 only marginally favored nontruncating variants (c) and had more variants around the 161 and 167 hotspots compared to cluster 1 (d). PPGL, pheochromocytoma/paraganglioma.

The characteristics of the two patient clusters (*K* = 2) are visually summarized in Figure 7. Patient cluster 1 (*N* = 1548) predominantly contained patients with non‐PPGL manifestations, where PPGL only accounted for 6% of the total phenotype counts (Figure 7a). Patient cluster 2 (*N* = 598) was PPGL dominant, accounting for 65% of the total phenotype count and present in every patient in the group (Figure 7a). Because only phenotype information was used in clustering, the following genotype statistics appear naturally and any apparent trends between cluster groups are influenced by VHL genotype‐phenotype manifestations, rather than the spectral clustering itself. Cluster 2 had marginally more variants in the α‐domain than the β‐domain, whereas cluster 1 had more variants in the β‐domain (Figure 7b). Both clusters have few variants outside these regions. Cluster 2 also heavily favored nontruncating variants over truncating, accounting for 94% of the variants in the cluster, whereas cluster 1 only marginally favored nontruncating variants (Figure 7c). Finally, cluster 2 tended to have more variants around the 161 and 167 hotspots than cluster 1 (Figure 7d).

**Figure 8:**
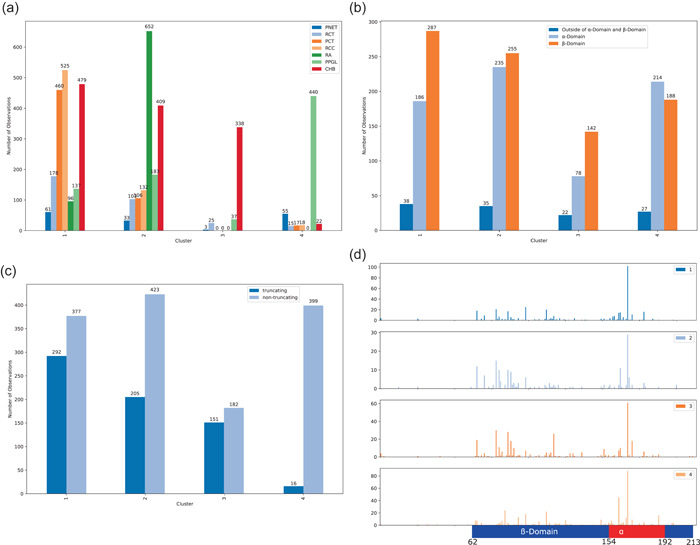
Cluster phenotype (a), variant type (b), variant domain (c), and codon statistics (d) for each of the four patient clusters. Patient cluster 1 was CHB, RCC, and PCT dominant, patient cluster 2 was RA and CHB dominant, patient cluster 3 was CHB dominant, and patient cluster 4 was PPGL dominant (a). Clusters 1, 2, and 3 had the majority of variants in the β‐domain compared to α‐domain, whereas the opposite trend was present in cluster 4 (b). Clusters 1 and 2 had more nontruncating than truncating variants, cluster 4 had vastly more nontruncating than truncating variants, and cluster 3 had a roughly equal distribution of truncating and nontruncating variants (c). Clusters 2 and 4 had more variants in α‐domain hotspots, especially codon 167, whereas clusters 1 and 3 had variants distributed more across the β‐domain (d). CHB, CNS hemangioblastoma; CNS, central nervous system; PCT, pancreatic cysts or tumors; PPGL, pheochromocytoma/paraganglioma; RCC, renal cell carcinoma.

Spectral clustering with *K* = 4 patient groups further explores relationships between phenotype trends and these relationships are visually summarized in Figure 8. Patient cluster 1 (*N* = 716) predominantly contained patients with PCT, RCC, and CHB, with fewer patients with RCT, PPGL, RA, and PNET (Figure 8a). In cluster 2 (*N* = 652), RA was dominant and was present in every patient in this group, with CHB also present in high amounts. Phenotypes RCT, PCT, RCC, PPGL, and PNET were seen in low amounts. Cluster 3 (*N* = 301) contained almost entirely patients with CHB, with every patient having CHB and very few having RCT and PNET. Finally, every patient in cluster 4 (*N* = 477) had PPGL, with fewer patients having PNET and CHB; even fewer patients had RCT, PCT, and RCC. The phenotype PPGL was prevalent in most of the clusters, besides cluster 3. Next, the genotype information for each cluster was analyzed. Cluster 1 (RCC, CHB, and PCT dominant) and cluster 3 (CHB dominant) had similar pVHL domain variant distributions (Figure 8b), favoring the β‐domain over the α‐domain. Cluster 2 (RA and CHB dominant) was almost equal in its α‐domain and β‐domain distributions. Cluster 4 (PPGL dominant) favored the α‐domain over the β‐domain. Looking at variant types (Figure 8c), cluster 3 (CHB dominant) had the highest truncating to nontruncating ratio, with slightly more truncating variants than nontruncating. Cluster 1 (RCC, CHB, PCT dominant) had the next highest ratio, having similar amounts of nontruncating and truncating variants. Cluster 2 (RA and CHB dominant) had mostly nontruncating variants, but still was about 30% truncating. Cluster 4 (PPGL dominant) was almost entirely nontruncating variants. Finally, looking at the codon positions of variants (Figure 8d), cluster 2 (RA and CHB dominant) and cluster 4 (PPGL dominant) were similar in that they both had a large amount of variants in the 167 hotspot and relatively fewer variants earlier in the gene. Cluster 4, however, also had a large hotspot at codon 161 where cluster 2 did not. Cluster 1 (PCT, RCC, CHB dominant) had many more variants earlier on in the gene and had a less prominent hotspot at 167. Cluster 3 (CHB dominant) generally had much fewer nontruncating variants and did not have any predominant hotspots.

## 4 DISCUSSION

### 4.1 Database curation

Our study is the first to conduct standardizing and harmonizing of germline genetic information in the literature, which will provide the foundation to utilize this fully in the management of patients. These types of studies will feed into larger initiatives such as ClinGen (Rehm et al., 2015) and our study serves as a model for the over 60 genes currently being curated with ClinGen.

Although the genetics of VHL has been well studied, the interpretation of *VHL* variants, in particular VUS, remains a common challenge in clinical genetics (Good et al., 2014). To overcome these challenges, we curated the largest aggregate population of VHL patients to date, with all published cases of VHL disease summarized and interpreted in the CIViC knowledgebase. While concordance on variant classifications is high within, but not across, individual laboratories (Amendola et al., 2016), agreement can be improved through collaborative review, shared knowledge and transparent discussions (Amendola et al., 2016; Good et al., 2014). The CIViC knowledgebase supports a congruent interpretation of *VHL* VUS through a standardized interface for knowledge sharing and interpretation. The CIViC knowledgebase is also completely open‐access and user‐friendly, making it a unique resource for VHL patients, families and community groups who can freely access information on variants and cancer‐types of interest. The raw data for all the VHL cases collected can also be accessed in an expanded datasheet (Supporting Information: Table S1) to support future investigations. Our research aims to reduce the burden on individual clinicians and investigators to independently review the literature for *VHL* gene variants and increase classification concordance by contributing to a collaborative approach in furthering our understanding of VHL disease. Finally, community curation on CIViC allows continuous uploading of newly available data, with new entries and discussions undergoing regular validation by trained CIViC editors. Ongoing efforts to continually update this data set are currently being undertaken in collaboration with the CIViC and ClinGen Community Curation working group (Rehm et al., 2015).

### 4.2 Large‐scale analysis

We analyzed data from 2146 patients, 1671 families, and 566 variants of the *VHL* gene. Overall, as expected, the findings from our analysis were consistent with previous VHL genotype–phenotype descriptions. As with other cohort reports, the prevalence of VHL disease did not seem to differ by sex. Of patients with available data, the mean last known age of patients was 33.4 years and the mean age of onset for VHL disease was 27.7 years, consistent with previous descriptions of early adulthood being the peak incidence of VHL disease (Maher, 1994; Maher et al., 2011). Furthermore, the results demonstrate that PPGL and RA had an earlier onset whereas RCC has a later age of penetrance, consistent with previous literature (Hes et al., 2005; Maher et al., 1990). Although approximately 20% of VHL cases have been suggested to arise de novo (F. M. Richards et al., 1995), our study only identified 72 of the 2117 patients with de novo variants (3.4%). A likely reason for this discrepancy is the criteria for de novo classification; previous definitions may have only included a “negative family history” but for the current study, both maternal and paternal genetic testing was required. This may support the importance of future work on germline mutations (for any gene study) to differentiate between a negative family genotype versus a negative family phenotype. The classification of VHL disease into VHL type 1 or type 2 was generally supported by the results, where 91% of patients with a PPGL had a nontruncating variant and this ratio of nontruncating to truncating variants was statistically different than all other phenotype distributions. Our findings also confirm and expand on hotspot variants previously described in VHL‐associated tumors and VHL patients (Hong et al., 2019; Stebbins et al., 1999). Among VHL patients, nine codons in the β‐domain (codons 65, 76, 78, 80, 86, 88, 98, 112, and 117) and five codons in the α‐domain (codons 161, 162, 167, 170, and 178) had a significantly elevated frequency compared to the other codons. Notably, peaks at codon 112 in the patient data were no longer significant in the family‐based analysis and two additional codons (codons 115 and 158) had a significantly elevated frequency in the family‐based analysis, accounting for larger families represented in the patient analysis. The reproducibility of previous reports increases our confidence that this aggregate database may be able to generate hypotheses and inform our knowledge about the general international VHL population.

We observed a great deal of similarities between PNET and PPGL. To our knowledge, genotypic similarities and correlations between PNET and PPGL have yet to be fully described in VHL disease. However, this observation may be considered intuitive when recognizing that both phenotypes are subtypes of neuroendocrine tumors. In a previous study of 31 VHL patients, PNET and pheochromocytomas were significantly associated with missense variants whereas pancreatic cysts were more frequent in patients with nonmissense variants (Fagundes et al., 2019). A larger population study describes a greater association of PNETs with intragenic variants compared to large deletions, with malignant PNETs specifically associated with VHL exon 3 variants with hotspots at 161 and 167 (Krauss et al., 2018; Tirosh et al., 2017). Likewise, PNETs have been described as more related to VHL type 2, defined by the presence of PPGL, compared to VHL type 1 (Igarashi et al., 2014). Our findings support this and suggest a select co‐occurrence between PNET and PPGL compared to other phenotypes. PNET and PPGL also favored nontruncating variants and variants in the α‐domain over the β‐domain, compared to other phenotypes; this may indicate a distinct and shared pathophysiology between PNET and PPGL and supports extending VHL type 2 to include PNET. This may be useful for clinicians predicting phenotypic outcomes for patients, for example increasing the clinical suspicion for PNET in VHL type 2 families. Although VHL screening guidelines are not currently differentiated based on a patient's genotype, our database serves as a critical starting point for more in‐depth genotype–phenotype predictions.

### 4.3 Machine learning analysis

Clustering was used for our data as this method excels at finding patterns in relationships between data points and does not require that all features be continuous or numerical. Patients connected within a single distinct cluster have the most optimized phenotype similarity. We can then assess these clusters, or each groups of patients, for distinct phenotype–genotype relationships. When clustering the patients in two groups, we observed that patients in cluster 1 had low occurrence of PPGL—corresponding to VHL type 1—and cluster 2 was PPGL dominant—corresponding to VHL type 2. When comparing the genotype characteristics between the clusters, we can appreciate that patients with nontruncating pathogenic variants in the α‐domain may be more likely to develop PPGL than other phenotypes. When clustering the patients in four groups, we observed cluster 4 as PPGL dominant with few patients also having PNET and CHB and low frequency of other manifestations. Patient cluster 1 was CHB, RCC, and PCT dominant and patient cluster 2 was RA and CHB dominant, although both clusters 1 and 2 still had some patients with other VHL manifestations, including PPGL and PNET. Interestingly, patient cluster 3 was CHB dominant with low frequency of all other manifestations. When reviewing the genotype patterns between these clusters, we can appreciate that clusters 1 and 3 have some similarities, with a higher frequency of truncating variants occurring more upstream of the *VHL* gene, in the β‐domain. This is clinically relevant considering that these clusters have high risks of RCC and CHB, which are VHL‐manifestations with the highest mortality rates (Binderup et al., 2017). Furthermore, these clusters can be used prospectively by clinicians to assess which phenotypes a patient with a specific genotype is most at risk for and inform surveillance strategies.

### 4.4 Limitations

The data set is entirely based on previously published literature to create an aggregated, comprehensive, and informative public resource. Papers written in non‐English were also excluded due to limited access to translators. As a result, the data are subject to publication bias where unpublished and non‐English cases are not captured, or conversely, some patients are over‐represented in the database. Although we resolved explicitly‐stated duplicated data, some cases were not always clear and may have been investigated by multiple groups without reference. If an author did not explicitly state the previous publication of a patient, there was no systematic way to accurately decipher duplicated data. To account for this, we engaged in multilevel analysis, where we investigated aggregated family‐based and variant‐based data that did not take individual patient numbers into account. By demonstrating that more conservative data types support similar conclusions, we can be more confident that our data are representative of the VHL population. When interpreting age of onset, we must also recognize the limitations of aggregating previously published data. For example, earlier papers contained information on clinical age at presentation of tumors, but with recently developing screening practices, more recent papers may report the age of onset when a patient's tumor is presymptomatic. Our work focused on germline *VHL* variants to create a useful clinical tool with research applications; however, we recognize that aggregated data on downstream genetic and metabolic events and epigenetics would be valuable for future investigations. Future work in data curation should expand beyond germline variants to further elucidate the role of all mechanisms of VHL disease progression.

Furthermore, while some authors published highly detailed case reports, other publications with different study scopes offered only a broad overview of their patient data set. By categorizing age data into three types and tagging cases as either “Patient,” “Family,” “Variant,” or “Tumor,” we attempted to limit the impact of heterogeneous data and strengthen the validity of our conclusions. For example, studies only reporting germline *VHL* variants in PPGL tumors, that did not specify whether other patient phenotypes were investigated, were tagged as “Tumor.”

### 4.5 Summary

We manually curated the largest open‐access database on VHL genotype and phenotype data, which can be accessed on the CIViC interface and through our Supporting Information: Tables. We demonstrated that our database is congruent with existing VHL knowledge and can be used to identify new patterns and associations in VHL disease, such as extending VHL type 2 classifications to PNET patients. We also demonstrated that our database can be used for unsupervised machine learning analyses. Our work will facilitate the sharing of VHL related information on a global scale and be accessible for use by clinicians, patients and researchers. This can also serve as a model for other genes and diseases with phenotypic heterogeneity.

## CONFLICT OF INTEREST

The authors declare no conflict of interest.

## Supporting information

Supporting information.

Supporting information.
