# Supplementary material for: Large scale genotype‐ and phenotype‐driven machine learning in Von Hippel‐Lindau disease
Source: Hum Mutat. 2022 May 10;43(9):1268–85. doi: 10.1002/humu.24392 (PMC9356987; doi:10.1002/humu.24392)
Supplement: Supplementary file 1 — Supporting information. [file HUMU-43-1268-s001.pdf]

Supplementary Information

Large scale genotype- and phenotype-driven machine learning in Von Hippel-Lindau disease

This Word file includes:

Supplementary Text
Supplementary References
Supp. Table S1 (caption)
Supp. Table S2 (caption)
Supp. Table S3
Supp. Table S4
Supp. Table S5
Supp. Table S6
Supp. Table S7

Supplementary Text

Supplemental Material and Methods

The BLOSUM62 and BLOSUM90 amino acid substitution matrices were used in the missense hotspot analysis, to account for some amino acid substitutions being more likely to occur than others. If the BLOSUM90 matrix is represented by $B90$, then element $B90_{ij}$ represents the log-odds that amino acid i will replace amino acid j, scaled in ½ bit units. For example, $B90_{AR} = -2$ means that alanine being substituted for arginine is $2^{\frac{-2}{2}} = \frac{1}{2}$ as likely than by chance. Conversely, $B90_{AA} = 5$ means that an alanine remaining unchanged is $2^{\frac{5}{2}} = 5.66$ as likely than by chance. Furthermore, a score like $B90_{AG} = 0$ indicates that change is $2^0 = 1$ times as likely as a random substitution. Since we are interested in the summation of missense mutations in hotspot codon locations, we wanted to make less likely substitutions count for more. To this end, we used the formula: $S_{ij} = 2^{-\frac{B90_{ij}}{2}}$ to calculate the score for an i to j amino acid substitution (for the BLOSUM90 matrix in this case). For each of the 213 codons, the scores S_{ij} for each mutation were summed together to produce one final count C_k for each codon k. The final counts C_k for $k = 1, 2, \ldots 213$ were used in the binomial test.

Clustering was used for our data as the publications from which our data was extracted did not always diagnose patients, families, or tumors with a VHL type classification, so fully-supervised learning techniques that require labels could not be used. Additionally, unlike deep-learning methods, clustering does not require larger quantities of data. If two patients show similar VHL phenotypes, then the edge between them is stronger. For phenotype resolution, we screened for all VHL phenotypes, therefore, there were no missing phenotypes and scores could be calculated between patients.

State-of-the-art spectral clustering methods use a graph-based approach that minimizes RatioCut, which attempts to find the lowest value of 'cuts' that separates nodes into distinct, unconnected subgraphs (Wei & Cheng, 1989). Here, "node" and "patient" are used interchangeably. If there are N patients, we represent the patient graph as $\boldsymbol{G} = (\boldsymbol{V}, \boldsymbol{E})$, where $\boldsymbol{V}$ corresponds to the set of all patients $\{x_1, x_2, \dots x_n, \dots x_N\}$, and $\boldsymbol{E}$ is the set of all edges $\{E_{1,2}, E_{1,3}, \dots E_{i,j}\}$, where each edge $E_{i,j}$ connects patient x_i to x_j. Edges in RatioCut clustering methods represent the nodes' affinities to one another, or how similar two nodes are. In our case, patients that have similar phenotypes should have higher edge values between one another. Furthermore, the phenotype properties of patients in our data are categorical rather than numerical, so we needed an affinity metric that operates on categorical data. Each patient x_n has a phenotype set $P_n = \{p_{CHB}, p_{PNET}, p_{RA}, p_{PPGL}, p_{RCT}, p_{PCT}, p_{RCC}\}$ where each variable $p_{phenotype}$ can only have values of 0 (if the phenotype did not manifest in that patient) or 1 (if the phenotype did manifest). We defined our patient affinity metric $E_{i,j}$ as the Jaccard index between two patient phenotype sets P_i and P_j:$E_{i,j} = \frac{|P_i \cap P_j|}{|P_i| + |P_j| - |P_i \cap P_j|}$. This produces a metric $0 \leq E_{i,j} \leq 1$ where a value of 1 means that patient x_i and x_j have identical phenotype manifestations. The edges are represented as an affinity matrix $\boldsymbol{E}$, where element i, j represents the phenotypic similarity between patients x_i and x_j.

To partition the patient graph via RatioCut, the normalized Laplacian matrix was needed (Wang et al., 2014). The Laplacian matrix is defined as $\boldsymbol{L} = \boldsymbol{I} - \boldsymbol{D}^{-\frac{1}{2}}\boldsymbol{E}\boldsymbol{D}^{-\frac{1}{2}}$, where $\boldsymbol{D}$ is a

network degree matrix where the total degrees of each node are on the diagonal elements, and the off-diagonal elements are set to 0.

Each patient x_n is associated with a label vector $y_n \in \{0,1\}^C$, so that $y_n(k) = 1$ if patient n belongs to the kth cluster of C total clusters, and $y_n(k) = 0$ otherwise. A partition matrix $\boldsymbol{Y} = (\boldsymbol{y_1^T}, \boldsymbol{y_2^T}, \ldots \boldsymbol{y_N^T})$ represents an entire patient labelling.

Then, the following optimization problem is solved to produce a patient partition:

$$\min Trace(\boldsymbol{Q}^T \boldsymbol{L} \boldsymbol{Q}) \;\; s.t. \boldsymbol{Q}^T \boldsymbol{Q} = \boldsymbol{I}$$

Scaled partition matrix $\boldsymbol{Q}$ is defined as $\boldsymbol{Q} = \boldsymbol{Y}(\boldsymbol{Y}^T \boldsymbol{Y})^{-\frac{1}{2}}$.

By minimizing the total score of the edge-cuts needed to partition the network into *C* distinct clusters, the patients connected within a single distinct cluster have the most optimized phenotype similarity. This revealed that each cluster, or each group of patients, roughly corresponded to a particular VHL subtype. We could then elucidate the phenotype-genotype relationship of each VHL subtype by comparing aggregated statistics of each cluster. We utilized patient data for clustering analysis because this data type had the highest sample size and relevance in interpretation. To create a metric for measuring phenotype similarity, the phenotypes associated with each patient, family, or variant had to be restricted to a singular true-or-false value (“is the phenotype present”) and therefore, our analysis did not consider the locations, sizes, or reoccurrences of any lesions.

Implementation of the above was done with Python 3.6. (Van Rossum, Hettinger, & Diedrich, 2008) The python libraries SciPy (Virtanen et al., 2020), NumPy (Harris et al., 2020), and Scikit Learn (Pedregosa et al., 2011) were all used.

All scripts used in analysis are available on GitHub at: https://github.com/RKteamVHL/VHL-Scripts

Supplementary References

Harris, C. R., Millman, K. J., van der Walt, S. J., Gommers, R., Virtanen, P., Cournapeau, D., . . . Oliphant, T. E. (2020). Array programming with NumPy. *Nature*, *585*(7825), 357-362. doi:10.1038/s41586-020-2649-2

Pedregosa, F., Varoquaux, G., Gramfort, A., Michel, V., Thirion, B., Grisel, O., . . . Duchesnay, E. (2011). Scikit-learn: machine learning in Python. *JMLR*, *12*, 2825-2830.

Van Rossum, G., Hettinger, R., & Diedrich, J. (2008). *The Python programming language*: Prentice Hall PTR.

Virtanen, P., Gommers, R., Oliphant, T. E., Haberland, M., Reddy, T., Cournapeau, D., . . . van Mulbregt, P. (2020). SciPy 1.0: fundamental algorithms for scientific computing in Python. *Nat Methods*, *17*(3), 261-272. doi:10.1038/s41592-019-0686-2

Wang, B., Mezlini, A. M., Demir, F., Fiume, M., Tu, Z., Brudno, M., . . . Goldenberg, A. (2014). Similarity network fusion for aggregating data types on a genomic scale. *Nat Methods*, *11*(3), 333-337. doi:10.1038/nmeth.2810

Wei, Y. C., & Cheng, C. K. (1989). Towards efficient hierarchical designs by ratio cut partitioning. *1989 IEEE International Conference on Computer-Aided Design. Digest of Technical Papers*, 298–301.

Supp. Table S1. Raw data, including information on genotype, phenotype and sample characteristics, extracted from 427 papers, representing 630 unique VHL variants, 2830 patients and 1986 families. Entries with the same ‘Kindred Case’ and ‘PMID’ are related. A unique kindred ID can be obtained by combining ‘Kindred Case’ with ‘PMID’. In cases where a family member was not genetically confirmed (but at least one other patient from that family was) the genotype information is reported as ‘unknown’. Large deletions will have an appropriate sequence ontology term (e.g. ‘deletion’ or ‘exon_loss_variant’) and the HGVS nomenclature fields as ‘unknown’, with more specific descriptions of the deletions available online through the CIViC knowledgebase.

Supp. Table S2. Extracted *VHL* variants that were identified as common population variants in gnomAD. These VHL cases were not included in our analysis. Entries with the same ‘Kindred Case’ and ‘PMID’ are related. A unique kindred ID can be obtained by combining ‘Kindred Case’ with ‘PMID’. In cases where a family member was not genetically confirmed (but at least one other patient from that family was) the genotype information is reported as ‘unknown’.

Supp. Table S3. Pairwise comparison of six generalized phenotypes (PCT, RCT, PPGL, RA, CHB, RCC) through a two-sample Kolmogorov–Smirnov test. Asterisks (**) indicate a significant result. PCT=pancreatic cysts or tumors, RCT=renal cysts or tumors, PPGL=pheochromocytoma/paraganglioma, RA=retinal angioma, CHB=CNS hemangioblastoma, RCC=renal cell carcinoma.

	RCC	RA	PPGL	CHB	RCT	PCT
**RCC**		alpha:0.00333333333333333335 ** p-value: 8.46196806848154e-07 D(35, 35): 0.6285714285714286	alpha:0.0033333333333333335 ** p-value: 2.4607538229304282e-11 D(35, 167): 0.6268605645851155	alpha:0.00333333333333333335 ** p-value: 0.0011125690129425436 D(35, 117): 0.36336996336996336	alpha:0.0033333333333333335 p-value: 0.26349876093136704 D(35, 7): 0.4	alpha:0.0033333333333333335 p-value: 0.25671506149952517 D(35, 19): 0.2736842105263158
**RA**			alpha:0.0033333333333333335 p-value: 0.9494638147088721 D(35, 167): 0.09084687767322498	alpha:0.00333333333333333335 ** p-value: 1.6894187562233043e-05 D(35, 117): 0.452014652014652	alpha:0.0033333333333333335 p-value: 0.44392343365385734 D(35, 7): 0.34285714285714286	alpha:0.0033333333333333335 ** p-value: 8.754258772825096e-05 D(35, 19): 0.6090225563909775
**PPGL**				alpha:0.00333333333333333335 ** p-value: 7.572916738141089e-10 D(167, 117): 0.3894774553457188	alpha:0.0033333333333333335 p-value: 0.301323528037018 D(167, 7): 0.35072711719418304	alpha:0.0033333333333333335 ** p-value: 1.69823135609537e-06 D(167, 19): 0.6073116924046643

**CHB**						alpha:0.0033333333333333335<br>p-value: 0.6430594813838395<br>D(117, 7): 0.2661782661782662	alpha:0.0033333333333333335<br>p-value: 0.10684592018882899<br>D(117, 19): 0.2878992352676563
**RCT**							alpha:0.0033333333333333335<br>p-value: 0.5420644572818486<br>D(7, 19): 0.3233082706766917
**PCT**							

Supp. Table S4. One-tailed binomial tests illustrate that codons with ≥21 or ≥17 missense variants for patients and families, respectively, were below the confidence interval of α=0.05 and the null hypothesis that the variants were distributed evenly was rejected for these codons. Asterisks indicate a significant result. PCT=pancreatic cysts or tumors, RCT=renal cysts or tumors, PPGL=pheochromocytoma/paraganglioma, RA=retinal angioma, CHB=CNS hemangioblastoma, RCC=renal cell carcinoma, PNET=pancreatic neuroendocrine tumor.

**Codon Position**	**p value**	**Corrected**	**Reject**	**Significance**
2	0.999718	0.000235	FALSE	ns
12	0.999718	0.000235	FALSE	ns
25	0.823188	0.000235	FALSE	ns
31	0.999718	0.000235	FALSE	ns
32	0.999718	0.000235	FALSE	ns
33	0.12292	0.000235	FALSE	ns
38	0.823188	0.000235	FALSE	ns
40	0.999718	0.000235	FALSE	ns
50	0.999718	0.000235	FALSE	ns

54	0.987954	0.000235	FALSE	ns
55	0.96216	0.000235	FALSE	ns
56	0.999718	0.000235	FALSE	ns
58	0.999718	0.000235	FALSE	ns
59	0.997409	0.000235	FALSE	ns
60	0.999718	0.000235	FALSE	ns
61	0.987954	0.000235	FALSE	ns
62	0.997409	0.000235	FALSE	ns
63	0.999718	0.000235	FALSE	ns
64	0.429119	0.000235	FALSE	ns
65	2.68E-28	0.000235	TRUE	****
66	0.999718	0.000235	FALSE	ns
68	0.038624	0.000235	FALSE	ns
70	4.18E-05	0.000235	TRUE	*
72	0.823188	0.000235	FALSE	ns
73	0.199337	0.000235	FALSE	ns
75	0.56882	0.000235	FALSE	ns
76	7.24E-10	0.000235	TRUE	****
77	0.999718	0.000235	FALSE	ns
78	3.83E-38	0.000235	TRUE	****
79	0.823188	0.000235	FALSE	ns
80	2.32E-14	0.000235	TRUE	****
81	0.302524	0.000235	FALSE	ns
82	0.009562	0.000235	FALSE	ns
84	3.87E-08	0.000235	TRUE	***
85	0.987954	0.000235	FALSE	ns
86	6.78E-31	0.000235	TRUE	****
87	0.823188	0.000235	FALSE	ns
88	1.60E-22	0.000235	TRUE	****
89	0.823188	0.000235	FALSE	ns
90	0.009562	0.000235	FALSE	ns
91	0.997409	0.000235	FALSE	ns
92	0.909418	0.000235	FALSE	ns
93	1.44E-05	0.000235	TRUE	*
94	0.009562	0.000235	FALSE	ns
95	0.997409	0.000235	FALSE	ns
96	0.12292	0.000235	FALSE	ns
97	0.987954	0.000235	FALSE	ns
98	3.71E-29	0.000235	TRUE	****
100	0.999718	0.000235	FALSE	ns
101	0.96216	0.000235	FALSE	ns
102	0.999718	0.000235	FALSE	ns
103	0.987954	0.000235	FALSE	ns

104	0.909418	0.000235	FALSE	ns
105	0.987954	0.000235	FALSE	ns
107	0.199337	0.000235	FALSE	ns
108	0.987954	0.000235	FALSE	ns
109	0.987954	0.000235	FALSE	ns
110	0.999718	0.000235	FALSE	ns
111	0.302524	0.000235	FALSE	ns
112	1.60E-22	0.000235	TRUE	****
113	0.12292	0.000235	FALSE	ns
114	0.001901	0.000235	FALSE	ns
115	0.071075	0.000235	FALSE	ns
116	0.96216	0.000235	FALSE	ns
117	4.73E-15	0.000235	TRUE	****
118	0.705775	0.000235	FALSE	ns
119	0.96216	0.000235	FALSE	ns
120	0.199337	0.000235	FALSE	ns
121	0.429119	0.000235	FALSE	ns
123	0.997409	0.000235	FALSE	ns
124	0.987954	0.000235	FALSE	ns
125	0.302524	0.000235	FALSE	ns
126	0.96216	0.000235	FALSE	ns
127	0.997409	0.000235	FALSE	ns
128	0.56882	0.000235	FALSE	ns
129	0.987954	0.000235	FALSE	ns
130	0.823188	0.000235	FALSE	ns
131	0.909418	0.000235	FALSE	ns
132	0.96216	0.000235	FALSE	ns
134	0.997409	0.000235	FALSE	ns
135	0.909418	0.000235	FALSE	ns
136	0.199337	0.000235	FALSE	ns
137	0.987954	0.000235	FALSE	ns
138	0.004377	0.000235	FALSE	ns
139	0.997409	0.000235	FALSE	ns
140	0.997409	0.000235	FALSE	ns
141	0.997409	0.000235	FALSE	ns
143	0.999718	0.000235	FALSE	ns
144	0.823188	0.000235	FALSE	ns
145	0.199337	0.000235	FALSE	ns
146	0.999718	0.000235	FALSE	ns
147	0.999718	0.000235	FALSE	ns
148	0.997409	0.000235	FALSE	ns
149	0.000785	0.000235	FALSE	ns
151	0.019775	0.000235	FALSE	ns

152	0.909418	0.000235	FALSE	ns
153	0.999718	0.000235	FALSE	ns
154	0.038624	0.000235	FALSE	ns
155	0.12292	0.000235	FALSE	ns
156	0.56882	0.000235	FALSE	ns
157	0.199337	0.000235	FALSE	ns
158	0.12292	0.000235	FALSE	ns
159	0.705775	0.000235	FALSE	ns
160	0.909418	0.000235	FALSE	ns
161	2.51E-101	0.000235	TRUE	****
162	3.46E-17	0.000235	TRUE	****
163	0.823188	0.000235	FALSE	ns
164	0.019775	0.000235	FALSE	ns
166	0.000116	0.000235	TRUE	*
167	0	0.000235	TRUE	****
168	0.429119	0.000235	FALSE	ns
169	0.997409	0.000235	FALSE	ns
170	9.42E-16	0.000235	TRUE	****
172	0.999718	0.000235	FALSE	ns
173	0.997409	0.000235	FALSE	ns
174	0.997409	0.000235	FALSE	ns
175	0.429119	0.000235	FALSE	ns
176	0.999718	0.000235	FALSE	ns
177	0.96216	0.000235	FALSE	ns
178	2.82E-09	0.000235	TRUE	****
179	0.999718	0.000235	FALSE	ns
180	0.997409	0.000235	FALSE	ns
181	0.999718	0.000235	FALSE	ns
183	0.909418	0.000235	FALSE	ns
184	0.823188	0.000235	FALSE	ns
185	0.705775	0.000235	FALSE	ns
186	0.999718	0.000235	FALSE	ns
188	0.000116	0.000235	TRUE	*
189	0.909418	0.000235	FALSE	ns
191	0.987954	0.000235	FALSE	ns
193	0.997409	0.000235	FALSE	ns
194	0.999718	0.000235	FALSE	ns
195	0.823188	0.000235	FALSE	ns
196	0.96216	0.000235	FALSE	ns
197	0.987954	0.000235	FALSE	ns
198	0.56882	0.000235	FALSE	ns
199	0.999718	0.000235	FALSE	ns
200	0.997409	0.000235	FALSE	ns

202	0.997409	0.000235	FALSE	ns
209	0.999718	0.000235	FALSE	ns
211	0.999718	0.000235	FALSE	ns

Supp. Table S5. The Chi-squared statistical test showed that there was a significant difference between truncating/non-truncating distributions between phenotypes. Asterisks indicate a significant result. PCT=pancreatic cysts or tumors, RCT=renal cysts or tumors, PPGL=pheochromocytoma/paraganglioma, RA=retinal angioma, CHB=CNS hemangioblastoma, RCC=renal cell carcinoma, PNET=pancreatic neuroendocrine tumor.

	**p value**	**Corrected**	**Reject**	**Significance**
('CHB', 'PCT')	0.026550126	0.002380952	FALSE	ns
('CHB', 'PNET')	9.28E-07	0.002380952	TRUE	***
('CHB', 'PPGL')	7.91E-57	0.002380952	TRUE	****
('CHB', 'RA')	0.000287979	0.002380952	TRUE	*
('CHB', 'RCC')	0.759188506	0.002380952	FALSE	ns
('CHB', 'RCT')	0.783367451	0.002380952	FALSE	ns
('PCT', 'PNET')	5.59E-09	0.002380952	TRUE	****
('PCT', 'PPGL')	3.24E-58	0.002380952	TRUE	****
('PCT', 'RA')	3.86E-07	0.002380952	TRUE	***
('PCT', 'RCC')	0.024777906	0.002380952	FALSE	ns
('PCT', 'RCT')	0.210298446	0.002380952	FALSE	ns
('PNET', 'PPGL')	4.73E-05	0.002380952	TRUE	*
('PNET', 'RA')	0.002855374	0.002380952	FALSE	ns
('PNET', 'RCC')	5.76E-06	0.002380952	TRUE	**
('PNET', 'RCT')	5.40E-06	0.002380952	TRUE	**
('PPGL', 'RA')	5.87E-32	0.002380952	TRUE	****
('PPGL', 'RCC')	6.00E-46	0.002380952	TRUE	****
('PPGL', 'RCT')	3.33E-38	0.002380952	TRUE	****
('RA', 'RCC')	0.004753614	0.002380952	FALSE	ns
('RA', 'RCT')	0.00491578	0.002380952	FALSE	ns

Supp. Table S6. The Chi-squared statistical test showed that there was a significant difference between α-domain and β-domain distributions across the phenotypes. Asterisks indicate a significant result. PCT=pancreatic cysts or tumors, RCT=renal cysts or tumors,

PPGL=pheochromocytoma/paraganglioma, RA=retinal angioma, CHB=CNS hemangioblastoma, RCC=renal cell carcinoma, PNET=pancreatic neuroendocrine tumor.

	p value	Corrected	Reject	Significance
('CHB', 'PCT')	0.967307927	0.002380952	FALSE	ns
('CHB', 'PNET')	6.94E-06	0.002380952	TRUE	**
('CHB', 'PPGL')	4.46E-09	0.002380952	TRUE	****
('CHB', 'RA')	0.03738373	0.002380952	FALSE	ns
('CHB', 'RCC')	0.948046328	0.002380952	FALSE	ns
('CHB', 'RCT')	0.523869654	0.002380952	FALSE	ns
('PCT', 'PNET')	4.97E-05	0.002380952	TRUE	*
('PCT', 'PPGL')	6.79E-06	0.002380952	TRUE	**
('PCT', 'RA')	0.118141763	0.002380952	FALSE	ns
('PCT', 'RCC')	1	0.002380952	FALSE	ns
('PCT', 'RCT')	0.637481347	0.002380952	FALSE	ns
('PNET', 'PPGL')	0.180272338	0.002380952	FALSE	ns
('PNET', 'RA')	0.001670541	0.002380952	TRUE	*
('PNET', 'RCC')	3.00E-05	0.002380952	TRUE	*
('PNET', 'RCT')	0.001028064	0.002380952	TRUE	*
('PPGL', 'RA')	0.001393976	0.002380952	TRUE	*
('PPGL', 'RCC')	1.26E-06	0.002380952	TRUE	***
('PPGL', 'RCT')	0.002145976	0.002380952	TRUE	*
('RA', 'RCC')	0.094668122	0.002380952	FALSE	ns
('RA', 'RCT')	0.493931698	0.002380952	FALSE	ns

Supp. Table S7. Although both PPGL and PNET phenotypes had more variants in the Elongin B and C binding site than the HIF-α binding site, none of the differences in functional site distributions were found to be significant. PCT=pancreatic cysts or tumors, RCT=renal cysts or tumors, PPGL=pheochromocytoma/paraganglioma, RA=retinal angioma, CHB=CNS hemangioblastoma, RCC=renal cell carcinoma, PNET=pancreatic neuroendocrine tumor.

	p value	Corrected	Reject	Significance
('CHB', 'PCT')	0.642817157	0.002380952	FALSE	ns
('CHB', 'PNET')	0.09144199	0.002380952	FALSE	ns
('CHB', 'PPGL')	0.073476573	0.002380952	FALSE	ns
('CHB', 'RA')	0.874493634	0.002380952	FALSE	ns
('CHB', 'RCC')	0.45397007	0.002380952	FALSE	ns

('CHB', 'RCT')	1	0.002380952	FALSE	ns
('PCT', 'PNET')	0.211287869	0.002380952	FALSE	ns
('PCT', 'PPGL')	0.396794551	0.002380952	FALSE	ns
('PCT', 'RA')	0.537348847	0.002380952	FALSE	ns
('PCT', 'RCC')	0.957462818	0.002380952	FALSE	ns
('PCT', 'RCT')	0.763561411	0.002380952	FALSE	ns
('PNET', 'PPGL')	0.451021601	0.002380952	FALSE	ns
('PNET', 'RA')	0.078657208	0.002380952	FALSE	ns
('PNET', 'RCC')	0.231811722	0.002380952	FALSE	ns
('PNET', 'RCT')	0.153657992	0.002380952	FALSE	ns
('PPGL', 'RA')	0.066216691	0.002380952	FALSE	ns
('PPGL', 'RCC')	0.446391773	0.002380952	FALSE	ns
('PPGL', 'RCT')	0.272152097	0.002380952	FALSE	ns
('RA', 'RCC')	0.373853085	0.002380952	FALSE	ns
('RA', 'RCT')	1	0.002380952	FALSE	ns
